# Pharmacological agonists for more-targeted CNS radio-pharmaceuticals

**DOI:** 10.18632/oncotarget.13418

**Published:** 2016-11-16

**Authors:** Luc Zimmer

**Affiliations:** Lyon Neuroscience Research Center, Université Claude Bernard Lyon 1 and Hospices Civils de Lyon, Lyon University Hospital, Lyon, France

**Keywords:** PET, radiopharmaceutical, serotonin, 5-HT_1A_ receptor, agonist

G-protein-coupled brain receptors provide abundant targets for psychotropic drugs and have been widely explored by PET (positron-emission tomography) brain imaging. PET is a nuclear medicine technique consisting in injecting molecules that have been radiolabeled with positron-emitters such as fluoride-18 or carbon-11. When such radiotracers can be implemented in animal models or humans, they are known as radiopharmaceuticals [1]. Around 800 G-protein-coupled receptors have been identified in the man, but specific PET radiopharmaceuticals are presently available for few of these potential therapeutic targets.

Developing a validated radiopharmaceutical enabling a brain receptor to be visualized and quantified involves a certain number of criteria, both radiochemical and radiopharmacological [2]. Chemically, the molecule in question, which is often a low-molecular-weight heterocycle, needs to be able to be radiolabeled: i.e., have an atom or chemical group that can be replaced by an atom of fluoride-18 or carbon-11. The short radioactive half-life of these isotopes (respectively, 110 and 20 minutes) requires a rapid radiolabeling method involving a minimal number of steps, validated pharmaceutically before injection under the PET camera. Radiopharmacologically, two main criteria are to be met: rapid brain penetration to enable quick acquisition after intravenous injection; and good specificity, with high affinity for the targeted receptor and very low affinity for any rival target or non-specific binding. If the fixation rate is low, with fewer than 5% of receptors occupied, such a “tracer” dose has to target a single protein.

**Figure 1:**
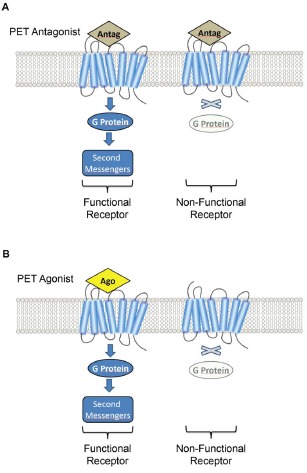
**A.** Schematic representation of PET antagonist (Antago) indiscriminately binding receptors in a G-coupled (functional) and uncoupled (non-functional) receptor state. **B.** The PET agonist (Ago) binds specifically to the G-coupled receptor state (functional receptors). Note that radiopharmaceuticals, at tracer dose, occupy only a fraction of the available receptors.

It is mainly because of this requirement of target-specificity that most PET radiopharmaceuticals developed to target receptors are, pharmacologically, antagonists. The range of antagonist molecules is wide, and many show satisfactory specificity and fixation suitable for PET brain imaging (k_on_ > k_off_). *In-vitro* pharmacologic studies, however, show that antagonists bind to both G-protein-coupled (i.e., functional) receptors and to non-functional non-G-coupled receptors of the same family [3]. Agonists, on the other hand, bind preferentially to G-protein-coupled (functional) receptors (Figure 1). The objective is to apply these pharmacologic findings to *in vivo* imaging, with the hypothesis that developing both an antagonist and an agonist radiopharmaceutical for the same receptor would enable two distinct cerebral fixation patterns, aimed at the same target, to be compared [4].

The serotoninergic system is especially well suited to this original radiopharmacologic strategy. 5-HT_1A_ family serotoninergic receptors are involved in numerous physiological (thermoregulation, respiration, sleep, memory, cognition, etc.) and pathological processes (mood disorder, anxiety, involuntary movement, apnea, etc.), making the 5-HT_1A_ receptor an interesting target for psychopharmacology and, more recently, neuropharmacology. Several PET 5-HT_1A_ radiopharmaceuticals ([^11^C]-WAY-100635, [^18^F]-FCWAY, [^18^F]-MPPF, etc.) are currently used in humans, but all are antagonists, with no “pure” agonists [5]. In collaboration with Neurolixis Inc, a biopharmaceutical company, we recently identified an agonist family with an *in-vitro* spectrum showing excellent specificity and affinity for the 5-HT_1A_ receptor. One of these, F13640, was radiolabeled by replacing a non-radioactive fluorine-19 by a radioactive fluorine-18, [^18^F]-F13640, with exactly the same chemical structure. Importantly, *in-vitro* autoradiographic studies showed that [^18^F]-F13640 binding was drastically reduced by pharmacologic uncoupling of the receptor from its G-protein, indicating the agonist behavior of this molecule [6]. Under the same experimental conditions, [^18^F]-MPPF, an antagonist of the same receptor, showed unchanged or slightly increased binding [6]. These autoradiographic experiments on human brain tissue provided encouraging results for this novel radiopharmacologic concept of targeting functional receptors by a radiolabeled agonist. Proof of concept, however, will only be provided by human *in-vivo* PET studies confirming the present animal model findings [7]: a radioactive agonist fixation pattern distinct from the radioactive antagonist fixation pattern in the same subject.

What are the possible applications of comparison between agonist and antagonist radiopharmaceutical patterns in humans? Here again, exploring 5-HT_1A_ receptors opens up the possibility of transposition to other neurotransmission systems. Our preliminary autoradiography studies on hippocampal tissue from Alzheimer patients [6, 8] showed that, in advanced disease on Braak staging, the fall-off in radioactive agonist binding was significantly earlier and stronger than for the reference antagonist, [^18^F]-MPPF. In other words, imaging the agonist revealed uncoupling (i.e., loss of 5-HT_1A_ receptor function) at early stages. At the same Braak stages, on the same PET brain slices, antagonist fixation was more sensitive to overall receptor density reduction, a sign of more advanced neurodegeneration. This allows functional receptor remodeling to be tracked at pre-dementia stages, opening up the possibility of better pathophysiological understanding, differential diagnosis or assessment of the impact of procognitive therapy. Another application could be for longitudinal assessment of the proportion of functional 5-HT_1A_ receptors with aging. Such neurophysiologic exploration could provide reference values for comparison between matched groups in psychiatry (schizophrenia, depression, etc.) or neurology (Parkinson's, etc.). Finally, this radiopharmacological strategy could prime research in pharmacoresistance: does a fall in the number of functional receptors correlate with reduced or abolished response to the pharmacotherapy targeting the receptor? In short, an “old pharmacological concept” deployed in molecular and functional neuroimaging opens up broad fields for new *in-vivo* targeting.
